# Xanthogranulomatous inflammation of the perimetrium with infiltration into the uterine myometrium in a postmenopausal woman: a case report

**DOI:** 10.1186/1472-6874-14-82

**Published:** 2014-07-15

**Authors:** Tomoko Inoue, Katsutoshi Oda, Takahide Arimoto, Taiki Samejima, Yutaka Takazawa, Daichi Maeda, Masashi Fukayama, Kei Kawana, Yutaka Osuga, Tomoyuki Fujii

**Affiliations:** 1 Department of Obstetrics and Gynecology, Faculty of Medicine, The University of Tokyo, 7-3-1 Hongo, Bunkyo-ku, Tokyo 113-8655, Japan; 2 Department of Pathology, The University of Tokyo, 7-3-1 Hongo, Bunkyo-ku, Tokyo 113-8655, Japan

**Keywords:** Xanthogranulomatous inflammation, Myometrial infiltration, Perimetritis

## Abstract

**Background:**

Xanthogranulomatous inflammation is an uncommon form of chronic inflammation that is destructive to the normal tissue of affected organs. Although xanthogranulomatous endometritis and xanthogranulomatous salpingitis of the female genital tract has been described previously, to the best of our knowledge, this is the first report of xanthogranulomatous inflammation with infiltration into the uterine myometrium from the perimetrium without endometritis.

**Case presentation:**

A 68-year-old Japanese woman with intermittent lower abdominal pain and low-grade fever who was initially treated with antibiotics underwent hysterectomy due to abscess formation in the posterior wall of the myometrium and perimetrium (the outer serosal layer of the uterus). Histopathological findings revealed that the abscess was caused by xanthogranulomatous inflammation with the granulation tissue and chronic inflammatory cells that consisted of focal and sheets of foam cells. The inflammation destroyed the perimetrial elastic lamina, and the myometrium was deeply infiltrated by the xanthoma cells. Neither endometritis nor salpingitis was coexistent with the xanthogranulomatous inflammation.

**Conclusion:**

The patient was diagnosed as xanthogranulomatous inflammation, most likely arising from the perimetrium. Our findings suggest that the perimetrium, as well as the endometrium and adnexae, is one of the origins of xanthogranulomatous inflammation in female genital tract.

## Background

Xanthogranulomatous inflammation (XGI) is an uncommon form of chronic inflammation that may destruct the normal tissue of affected organs. Histopathologically, it is characterized by a marked proliferative fibrosis, parenchymal destruction, and infiltration of foamy histiocytes intermixed with other inflammatory cells.

XGI has been reported in multiple organs, most commonly in the kidney and gall bladder, and less commonly in the female genital tract [1,2]. XGI in the female genital tract has been primarily observed as endometritis and/or salpingitis (tubo-ovarian abscess) [3-12]; XGI with uterine myometritis but without endometritis has not been reported. Herein, we report a case of a 68-year-old woman diagnosed with XGI with myometritis, in whom the histopathological findings revealed that XGI was located only in the myometrium and perimetrium, without affecting the endometrium.

## Case presentation

A 68-year-old (gravida 4, para 2) post-menopausal woman presented to our hospital with vaginal bleeding. She had a history of multiple uterine fibroids over the past 20 years, but no history of endometriosis, pelvic inflammatory disease, use of an intrauterine device, or surgery. Her family history was unremarkable.

**Figure 1:**
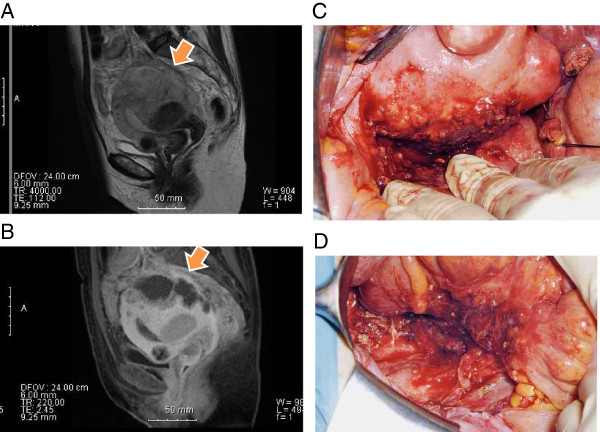
**Magnetic resonance imaging (MRI) and photographs during surgery.** MRI findings of a 6 cm mass in diameter (arrow) with high-intensity by sagittal T2-weighted **(A)** and low-intensity by sagittal T1-weighted (fat-suppressed) imaging, combined with gadolinium-enhanced imaging **(B)**, located in the posterior myometrium. **(C, D)** Intra-pelvic adhesions secondary to perimetrial inflammation. Adhesions around the posterior wall of the uterus **(C)** and sigmoid colon **(D)**.

**Figure 2:**
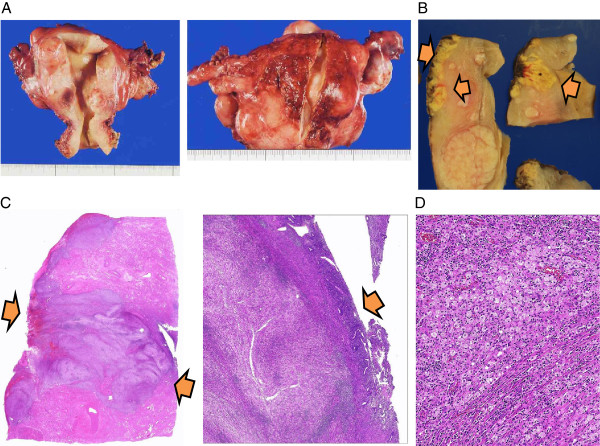
**Macroscopic and microscopic features of the abscess. (A)** Photograph of the excised uterus. The endometrium was smooth with no inflammatory changes (left), and the abscess was located in the posterior wall of the uterus (right). **(B)** Photograph of the cut surface of the uterus. The posterior myometrium and perimetrium were yellowish and with areas of necrosis and hemorrhage. **(C)** Microscopic features (low power): The inflammation destroyed the perimetrium, infiltrated deep into the myometrium (left) but did not reach the endometrium (right). **(D)** Microscopic features (low power): The xanthogranulomatous inflammation with foamy histiocytes and neutrophils, infiltrating into the myometrium. The smooth muscle cells were dispersed diffusely among the inflammatory cells.

Ultrasonography revealed no abnormal masses except for multiple uterine fibroids. Cytoscreening of the cervix and endometrium was performed. One week later, the patient presented to our hospital with a fever (up to 39°C) and abdominal pain. *Escherichia coli* was detected in her vaginal culture. She was diagnosed with endometritis and treated with antibiotics (levofloxacin). In addition, the cytology of the endometrium showed atypical columnar cells with inflammation, for which further examination was recommended to rule out malignancy. After her symptoms improved, endometrial biopsy was performed under levofloxacin. Pathologically, inflamed endocervical mucosa and endometrium were observed, but no malignant cells were detected. The white blood cell count was 8.3 × 10^3^/uL. Six weeks later, she visited our hospital again due to persistent low-grade fever and abdominal pain. Transvaginal ultrasonography revealed an irregularly shaped mass, 6 cm in diameter, in the posterior wall of the myometrium. Contrast-enhanced computed tomography (CT) scan suggested an intramural abscess besides multiple uterine fibroids. Magnetic resonance imaging (MRI) also revealed an irregularly shaped mass, 6 cm in diameter, which was localized in the posterior wall of the uterus (Figure 1A and B). On T2-weighted MRI, the perimetrium of the posterior wall was revealed to be edematous with suspected adhesions to the sigmoid colon and rectum. The white blood cell count and C-reactive protein (CRP) were elevated to 20.2 × 10^3^/uL and 14.6 mg/dL, respectively.Total abdominal hysterectomy and bilateral salpingo-oophorectomy were performed. The uterus was enlarged due to the uterine fibroids and abscess, and dense adhesions existed around the posterior wall of the uterus and sigmoid colon (Figure 1C and D). There was neither inflammation nor enlargement in the bilateral adnexae. The postoperative course was uneventful, and the patient was discharged 8 days after the surgery. An endoscopic examination of the rectum and colon showed no evidence of diverticulum and malignancy.Macroscopically, an abscess, 6 cm in diameter, was present in the posterior perimetrium (Figure 2A). The cut surface of the abscess was purulent with hemorrhage, necrosis, and cystic degeneration, and was apart from the endometrium (Figure 2B). Microscopically, marked infiltration of foamy histiocytes with clear lipid-containing cytoplasm, together with abundant lymphocytes and plasma cells, was observed in the posterior uterine myometrium. The infiltration destroyed the perimetrial elastic lamina and the myometrium was deeply infiltrated by the granulation tissue and chronic inflammatory cells with focal or sheets of foam cells (xanthoma cells) (Figure 2C and D). Neutrophils were also observed at the posterior wall surrounding the abscess cavity. These findings were consistent with XGI of the uterine corpus. However, there were no xanthoma cells in the endometrium and bilateral adnexae. Thus, we diagnosed the patient with XGI arising from the perimetrium and infiltrating deep into the posterior uterine myometrium.

## Discussion

XGI is a rare inflammatory disease, which may affect various organs, including the kidney and gallbladder [1,2]. XGI is a severe inflammation and might be lethal in some cases [7]. So far, all reported XGI cases involving the female genital tract have been of endometritis and salpingitis [3-12]. As the perimetrium has not been previously reported as the origin of XGI, making a diagnosis in this case required careful consideration. Although inflammation did not affect the endometrium and adnexae, it is still possible that XGI from previously experienced endometritis infiltrated from the endometrium to the uterine myometrium and perimetrium. Indeed, her first endometrial cytology and biopsy indicated inflamed endometrial cells. In addition, it is difficult to exclude the possibility that the sigmoid colon may be an origin of XGI because antibiotics had been administered before the hysterectomy. However, the microscopic spread pattern of XGI in this case suggests that the origin is more likely to be the perimetrium rather than the endometrium, fallopian tubes, or sigmoid colon.

One of the primary differential diagnosis of XGI is malignancy [8,13]. Indeed, endometrial hyperplasia and endometrial carcinomas have been detected in patients with XGI with endometritis [14]. Therefore, cytological and histological diagnosis is important to rule out endometrial cancer in XGI cases. Although it is not clear whether the initial inflammation in the endometrium was associated with the abscess formation, the XGI might be affected by intrauterine examination in this case. Further study is warranted to clarify the etiology of XGI in the genital tract.

## Conclusion

Here, we have reported a case of severe XGI, originating from the perimetrium into the deep posterior uterine myometrium. Our case highlights the need for careful assessment of the inflammatory diseases in female genital tract. Pelvic inflammatory diseases are commonly associated with sexually transmitted diseases, intrauterine devices, or previously experienced surgeries, and the inflammations commonly originate from the adnexae and the endometrium. However, our case indicated that the inflammation of the perimetrium might deeply infiltrate into the myometrium and cause dense adhesion with the colorectum. Thus, careful considerations are warranted to diagnose the origin of the inflammatory diseases and to appropriately treat the diseases in the female genital tract.

### Consent

Written informed consent was obtained from the patient for publication of this Case report and any accompanying images.

## Abbreviations

XGI: Xanthogranulomatous inflammation; CT: Computed tomography; MRI: Magnetic resonance imaging; CRP: C-reactive protein.

## Competing interests

The authors declare that they have no competing interests.

## Authors’ contributions

All authors have made substantial contributions to the diagnosis and treatment of this case. TI and KO: treated the case and wrote the manuscript. YT, DM and MF diagnosed the case pathologically. TS, TA, KK, YO, and TF contributed to the diagnosis, obtained informed consent, and determined the management of the case. All authors read and approved the final manuscript.

## Authors’ information

Drs. TI (MD) and TS (MD) are obstetricians and gynecologists, and are graduate students of “Graduate Scool of Medicine, The University of Tokyo”. Drs KO (MD, PhD) and KK (MD, PhD) are gynecologic oncologists, and are associate professors of “Department of Obstetrics and Gynecology/Graduate School of Medicine, The University of Tokyo”. Dr. TA is a gynecologic oncologist, and a project assistant professor of “Department of Obstetrics and Gynecology, The University of Tokyo” Drs. YT (MD, PhD) and DM (MD, PhD) are pathologists, who have a specialty of gynecologic pathology. Dr. MF (MD, PhD) is a professor of “Department of Pathology, The University of Tokyo”. Drs. YO (MD, PhD) and TF (MD, PhD) are professors of “Department of Obstetrics and Gynecology/Graduate School of Medicine, The University of Tokyo”.

## Pre-publication history

The pre-publication history for this paper can be accessed here:

http://www.biomedcentral.com/1472-6874/14/82/prepub
